# Understanding Eye Movement Signal Characteristics Based on Their Dynamical and Fractal Features

**DOI:** 10.3390/s19030626

**Published:** 2019-02-01

**Authors:** Katarzyna Harezlak, Pawel Kasprowski

**Affiliations:** Institute of Informatics, Silesian University of Technology, Akademicka 16, 44-100 Gliwice, Poland; pawel.kasprowski@polsl.pl

**Keywords:** eye tracking, infrared oculography, chaotic dynamics, fractal theory, filtering methods

## Abstract

Eye movement is one of the biological signals whose exploration may reveal substantial information, enabling greater understanding of the biology of the brain and its mechanisms. In this research, eye movement dynamics were studied in terms of chaotic behavior and self-similarity assessment to provide a description of young, healthy, oculomotor system characteristics. The first of the investigated features is present and advantageous for many biological objects or physiological phenomena, and its vanishing or diminishment may indicate a system pathology. Similarly, exposed self-similarity may prove useful for indicating a young and healthy system characterized by adaptability. For this research, 24 young people with normal vision were involved. Their eye movements were registered with the usage of a head-mounted eye tracker, using infrared oculography, embedded in the sensor, measuring the rotations of the left and the right eye. The influence of the preprocessing step in the form of the application of various filtering methods on the assessment of the final dynamics was also explored. The obtained results confirmed the existence of chaotic behavior in some parts of eye movement signal; however, its strength turned out to be dependent on the filter used. They also exposed the long-range correlation representing self-similarity, although the influence of the applied filters on these outcomes was not unveiled.

## 1. Introduction

Many biological objects and physiological phenomena are perceived as complex dynamical systems, characterized by irregular and nonstationary properties. Detection of these hidden, important features is a complicated task, because of a lack of clearly defined rules describing their characteristics. Such knowledge may be gained by system observations that produce a biological time series, exploration of which requires appropriate techniques. Very often, methods of nonlinear time series analysis are chosen for this purpose, which enable investigating both a system’s structure and its dynamics. For example, analysis of an electroencephalogram (EEG) signal was conducted in [1,2], while characteristics of electrocardiogram (ECG) signals were explored in [3,4]. Another piece of research was devoted to gait stability assessment, which is presented in [5,6].

When studying biological system characteristics, one may encounter variance among the measurements obtained for a particular object, despite keeping the experiment’s conditions as identical as possible. This variance may result from the noise introduced by an experimental measuring setup, but also from the intrinsic variability observed in the output signals of biological systems, caused by background fluctuations originated, for example, in brain functions and either at neuronal or muscle levels.

### 1.1. Eye Movement Basics

Eyes and the oculomotor system, responsible for managing the muscles moving the eyes, belong to the group of the above-mentioned biological systems. A time series, obtained by an observation of this system, is a set of eye positions consisting of two main eye movement events: fixations and saccades [7]. Fixations are recognized, when the eyes are almost stable gathering information regarding a scene. The average fixation duration amounts to 200–300 ms; however, they may last from tens ms up to several seconds. Saccades are very rapid movements, during which the eyes change a position to reach another fixation point. During this movement, no information is acquired. A saccade is described by three attributes: (1) amplitude—the size of the saccade, which may vary between $4^{\circ }$ and $20^{\circ }$; (2) duration—the time taken to complete the saccade, most saccades are complete within a few tens of milliseconds: 30–80 ms; (3) velocity, which may assume values from $30^{\circ }$/s reaching up to even $500^{\circ }$/s. Additionally, within the fixation, some micro-movements are observable, which are responsible for centering fixations and preventing the fading of an image of an observed scene [8,9,10]. Within this group, microsaccades, drifts, and tremors may be listed. A microsaccade is a motion with a small amplitude (10–40${}^{\prime }$), velocity of 15–50${}^{\circ }$/s and duration between 10 and 30 ms. Tremors are high-frequency (40–100 Hz) movements with very low amplitudes (approximately $0.0017^{\circ }$) and a peak velocity of 20${}^{\prime }$/s. The last fixation component—drift—is a slow, smooth movement (6–25${}^{\prime }$/s) of lower frequency (less than 40 Hz) [7]. Because of these micro-movements, a fixation cannot be related to one point and some additional measures must be involved, including: the size of spatial dispersion and velocity of eye movement during a fixation.

A time series, representing an eye movement signal, is collected with the usage of a specialized device called an eye tracker. For modern eye trackers, it is feasible to register such a signal with a sampling rate ranging from 30 Hz up to 2000 Hz and for the analysis of the system dynamics the higher the sampling rate the better.

### 1.2. The State of The Art

A great deal of effort has been put into understanding the biology of the eye and the nature of its movements. Recently, several steps have been undertaken to unveil the characteristics of eye movements, perceived as representatives of biological nonlinear dynamic signals. Many of these reveal features of chaotic behavior; in which a breakdown or reduction may indicate stress to the system or pathology [11]. A few studies concerning eye movements have been conducted in this field. The authors of [12,13], based on the analysis of eye-gaze locations, confirmed the presence of dynamics expressing deterministic chaotic behavior. The former research was aimed at revealing the dynamics type, which characterizes behavior of saccadic neural system. Eye movements were recorded with a 1000 Hz sampling rate during a simple visually guided saccade test and one with a cognitive load. Based on the registered signal, several variables describing a nonlinear dynamic system were evaluated. They were: the Largest Lyapunov Exponent (LLE), the correlation dimension and the autocorrelation represented by the Hurst exponent. They were estimated for raw and smoothed data; however, the smoothing method was not described. A dominant chaotic trend was shown to underline the dynamics of the neural system responsible for saccadic eye movement. The limitation of this work is the involvement of only one participant, which means that the obtained results cannot be treated as reference data. The purpose of the second mentioned study was to explain eye movement characteristics during a visual search. The First Lyapunov Exponent (FLE) and the attractor plot were evaluated as a function of layout complexity of the display. Ten young men took part in the experiment. Eye movement data were collected with the usage of the device, which enabled recording signals with a 60 Hz frequency. The obtained positive values of the FLE showed that the time series of eye-gaze locations included chaotic property. However, some doubts arise when the data sampling rate is taken into account. It could possibly be insufficient to uncover the full dynamics of eye movement signal. According to [7] a saccade shorter than 10${}^{\circ }$ cannot be recognized accurately with a sampling rate equal to 60 Hz. Thus, for a saccade analysis, eye trackers with sampling rate equal to or greater than 200 Hz should rather be used. A chaotic dynamic nature was also revealed in [11] regarding the accommodative process invoked to change the focus on an object placed at different distances. Additionally, arguments for chaos existence in eye movement were shown in [14,15,16] when exploring its velocity during prolonged fixations. Eye movement signals were registered with a sampling rate equal to 1000 Hz during an experiment when participants were expected to follow a point appearing in various locations on the screen. Twenty-four participants took part in this experiment. For each point presentation—relating to one fixation—one time series was defined. Before subjecting to further analysis, a low-pass filter with a 50 Hz cut-off frequency was applied regarding each time series. In the first two studies analysis of the LLE was conducted, while in the third one, correlation dimension and entropy were explored. These calculations regarded the original and filtered signals, yet only one filter type was applied with a fixed cut-off frequency.

The problem of noise is inevitably related to the eye movement measurement process and it has been investigated in eye tracking studies to recognize its shape, strength and to ascertain its importance as well as to alleviate its influence on eye movement processing [17]. An interesting review and studies in this field, aimed at the comparison of various real-time filters designed to denoise eye movements gained from low-sampling devices are presented in [18]. Komogortsev and Khan [19] applied the Kalman filter for smoothing gaze path. In another piece of research [20] the Recursive Online Weight Average filter (ROWA) was proposed to remove and replace noisy data obtained from the eye tracker in such a way that it can remove noise from corrupted signals and replace it with the best approximation, while leaving the remaining signals untouched. A comparison of this method with other filter types was also conducted. Warman et al. [21] compared three signal processing filters, namely Moving Average, Gaussian, and Kalman filters to remove noise in smooth pursuit eye movements. Additionally, the analysis of a filter’s influence on building an eye movement model was presented in [22]. Two median filter types: basic median and AOI (Area of Interest) median were applied. The latter calculates a median for all subsequent points related to the same target. Both kinds of methods were used regarding various eye movement datasets.

The most important goal of the above-mentioned studies was to obtain the best possible adjustment between people’s gazes and the observed targets [7,22]. However, none of the conducted research was aimed at examining the impact an applied filter has on reconstructing eye movement dynamics. The exploration of this influence is one of the main objectives of the presented studies. This issue seems to be an important topic, as a properly described eye movement nature may prove useful in differentiating healthy systems and those with disease. Furthermore, it may be used in eye movement events detection.

### 1.3. Paper’s Contribution

In the presented research, attention was focused on examining features of chaotic dynamics and the existence of self-similarity in eye movement signals to provide a description of young, healthy, oculomotor system characteristics. Additionally, in both the analyzed scopes the dependency of the revealed characteristics on the used filter was checked. Analysis was conducted regarding the data recorded by the eye tracker using infrared oculography, during an experiment based on the ’jumping point’ paradigm. The research purpose was realized by means of the time series analysis and embedding theory and the exploration was conducted with several defined time series segments. To the best of the authors’ knowledge, such studies are the first in this area, thus the research contribution may be defined as follows:

- introduction of a new set of intervals for analyzing eye movement time series representing one fixation,
- assessment of chaotic eye movement behavior in the defined scopes, taking different filtering methods into account,
- fractal analysis aimed at ascertaining long-range correlation existence in an eye movement signal corresponding to one fixation.

## 2. Material and Methods

This section is divided into three parts. Theoretical basics are provided in the first one. The second part is devoted to the presentation of the experimental data set. Finally, the group of methods used in the studies, with applied parameters, is described.

### 2.1. The Embedding and Fractal Theory

As previously mentioned, the most common difficulty encountered when analyzing biological signals is the lack of state equations, which directly describe a system’s behavior. Consequently, it must be inferred from biological system observations—a sequence *O* of N scalar measurements obtained with a *T* sampling rate. Given such a series of observations, it is possible to reconstruct the dynamics of the original system. However, prior to this, the time series must be transformed into a time delay embedded one (*Y*), according to the following formula (Equation (1)):

(1) $$y\left(i\right)=\left[o\left(i\right),o\left(i+\tau \right),\cdots ,o\left(i+\left(m-1\right)\times \tau \right)\right],$$

where τ represents time delay—which is a multiplicity of *T*; *m* is the dimension of the Euclidean space, in which the time series state space is reconstructed; o(i) is an observation, $i=\left\{1,2\cdots ,M\right\}$ and M=N−(m−1)×τ [23].

The embedding parameters τ and *m* are unknown and must be evaluated for each analyzed time series. The first one is estimated based on the correlation present in the data, by calculating the mutual information factor [24]:

(2) $$I\left(\tau \right)=\sum_{h=1}^{j}\sum_{k=1}^{j}P_{h,k}\left(\tau \right)\;log_{2}\frac{P_{h,k}}{P_{h}P_{k}},$$

where $P_{h}$ and $P_{k}$ denote the probabilities that the variable takes the value inside the *h*th and *k*th intervals, respectively, and $P_{h,k}\left(\tau \right)$ is the joint probability that $x_{i}$ is taken from interval *h* and $x_{i}+\tau$ from interval *k*. The time delay τ is the first local minimum returned by I(τ).

For evaluating the second parameter, the false nearest neighbor method is frequently used [25], providing the dimension in which orbits—a path followed by a system—unfold from overlapping. This means that the ratio of distances between a pair of points—$y_{i}$ and its nearest neighbors $y_{i}^{NN}$—seen in dimensions m+1 and in dimension *m*, is less than a predefined threshold *R* (Equation (3)).

(3) $$R>\frac{\left|x_{i+(m+1)\tau }-x_{i+(m+1)\tau }^{NN}\right|}{∥y_{i}-y_{i}^{NN}∥}.$$

Reconstruction of the system’s states—the phase space—opens the possibility of exploring and identifying its dynamics. Systems that reveal a deterministic nature tend to evolve towards an *attractor*, the orbit attracting the system from various starting positions. One of the indicators describing system dynamic characteristics is the LLE, which is calculated by tracing neighboring points when the system develops. Negative values of this exponent indicate convergence, whereas positive demonstrate divergence and chaos. It can be evaluated according to the formula presented in Equation (4) [26]:

(4) $$\lambda =\frac{1}{i\Delta t}\ln\frac{d_{i}\left(i\right)}{d_{j0}},$$

where Δt is the sampling rate of the time series, $d_{j0}$ is the initial pair separation and $d_{i}\left(i\right)$ is the divergence between a *j*th pair of the nearest neighbors after *i* time steps. The λ represents LLE.

For analyzing signals, chaos theory also provides fractal methods, which enable assessment of the presence or absence of fractal properties representing self-similarity. Such a self-similarity may be reflected by a fractal dimension, as studied in [16], but also by investigating long-range correlations. The correlation structure of dynamic processes is usually explored by ascertaining the Hurst exponent, which points out the probability that after a process event, a similar occurrence will take place [27]. Values of this parameter range from 0 to 1: when lower than 0.5 it indicates anti-persistent processes, equal to 0.5 relates to independent observations—an uncorrelated signal (white noise) and finally, if greater than 0.5 denotes positive autocorrelation in the signal (pink or fractal noise characterized by the power law in the power spectral density). When this exponent takes the value 1.5, it represents Brownian noise (random noise produced by Brownian motion—random walk).

The most commonly used method for evaluating the Hurst exponent is detrended fluctuation analysis (DFA) [28]. This method has been successfully applied to such biological processes as heart rate dynamics, neuron spiking or human gait [29] and consists of several steps. According to this method, the previously integrated series y(k) is divided into non-overlapping intervals of length *n*. For each such segment, the linear approximation $y_{n}$—representing the trend in a given section—is calculated by use of the least-squares fit. The average fluctuation F(n) of the signal around the trend is subsequently evaluated based on the formula (Equation (5)):

(5) $$F\left(n\right)=\sqrt{\frac{1}{N}\sum_{k=1}^{N}{\left[y\left(k\right)-y_{n}\left(k\right)\right]}^{2}}.$$

The above-described steps are performed for several defined interval lengths and the dependency of fluctuation F(n) on *n* is analyzed. Typically, F(n) increases with interval length *n* according to the power law:

(6) $$F\left(n\right)\approx n^{\alpha },$$

where α is expressed as the slope of a double logarithmic plot of F(n) as a function of *n*. It is converted to the Hurst exponent *H* according to the following rules: H=α if α belongs to the range [0⋯1]; H=α−1 when α ranges between 1 and 2.

### 2.2. Dataset Description

**Figure 1:**
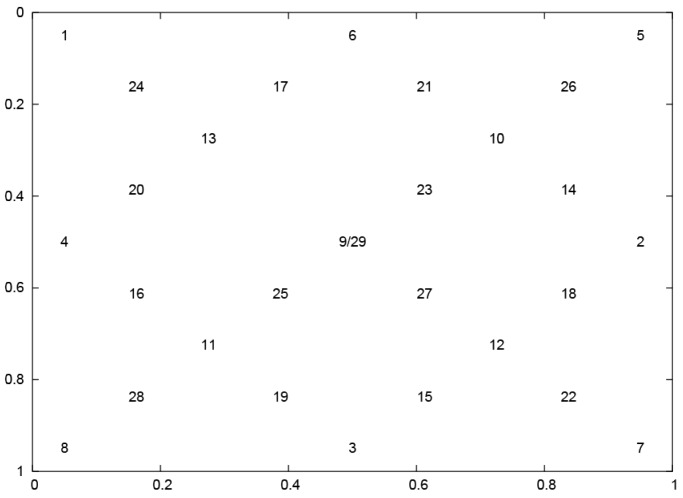
Layout of stimuli applied during experiments– based on the universal scale—the top–left corner of the screen is represented by (0.0, 0.0) coordinates and the bottom–right corner by (1.0, 1.0) respectively.

The research goal was realized with the use of data—(see *Supplementary Materials*)—collected from the 24 healthy participants, with normal vision, during two sessions, separated by a three-week break. The participants were expected to focus on a jumping point presented in the 29, various, evenly dispersed positions (Figure 1). Only one point at a time was presented on the screen, for 3 s.

**Figure 2:**
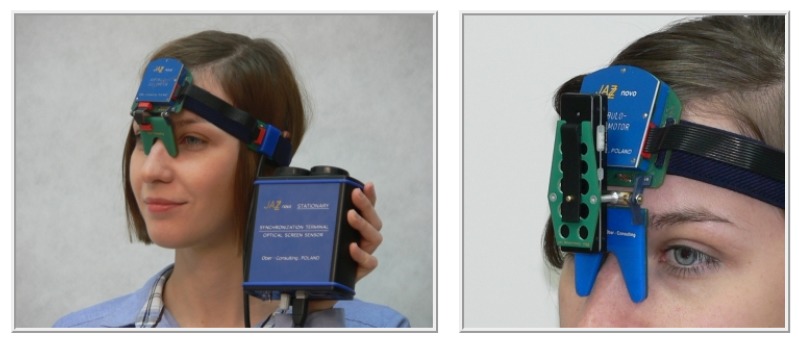
The head-mounted JAZZ-novo eye tracker [30] as used in the research.

Eye movements were registered with a 1000 Hz sampling rate by means of the head-mounted JAZZ-novo eye tracker [30]. It uses Direct Infra Red Oculography (IROG), which is embedded in the Cyclop ODS sensor measuring the resultant rotations of the left and the right eye. During the registration, eyes are illuminated with a low intensity infrared (IR) light. The difference between the amounts of IR reflected back from the eye surfaces, carries information pertaining to the eye position changes. Due to the location of optoelectronic transducers, placed between the eyes (thus hiding the sensor assembly behind the “shadow” of the nose), the Cyclops-ODS provides minimal intrusiveness (Figure 2).

**Figure 3:**
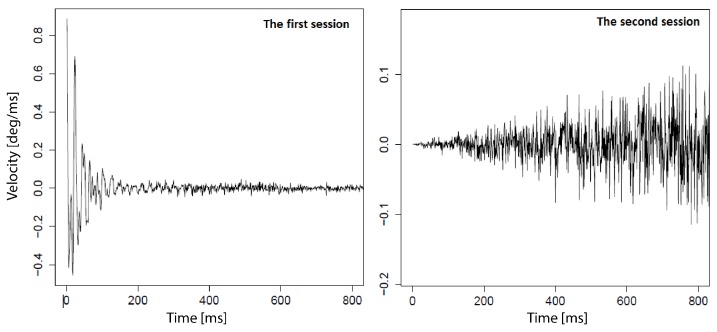
Examples of two time series registered for the same stimulus position and for one participant, yet during different sessions. The first session with the correct signal is shown on the left, while the signal registered during the second session with abnormal features is visible on the right.

All recordings gathered during one user’s session were divided into 29 sets—one per one fixation—including 3000 samples registered between stimulus position changes. Taking the number of participants and the two sessions into account, it gave in total: 29 × 24 × 2 = 1392 sets, further referred to by *N*. From the group of 1392 time series, 58 were excluded—for two people, from the second session of the experiment—as abnormal eye movement behavior was observed. It was probably introduced by the experiment environment, as in the first session no unexpected data was discovered for these participants. Thus, finally 1334 time series were analyzed. Examples of two time series registered for the same stimulus position and for one of the previously mentioned participants, yet during different sessions are presented in Figure 3.

### 2.3. The Methods Applied

All the aforementioned data sets were processed by a group of filters, among which three, different, frequently used methods were considered:

- the Running Median (RM); method replacing the middle element of the defined window—which moves by one element along the axis of the independent variable—with the middle value of the ordered list of window elements. Three window sizes were used: 5, 9, and 15—the resulting filtered observations are denoted by M5, M9, M15.
- the third degree Savitzky-Golay (SG) filter [31] with two window lengths: 7 and 15. The underlying signal within the moving window is approximated by a polynomial of the given order. A least-squares fit of consecutive data points (defined by window size) to a polynomial is performed. The calculated central point of the fitted polynomial curve is the new smoothed data point. Data sets after processing by this method are denoted in the subsequent analysis by SG7, SG15.
- the Daubechies’ wavelets (Db) [32]—a method denoising a signal by means of wavelet transform. It enables presentation of the signal in the form of a linear combination of two types of coefficients representing elements of low and high frequency. By setting the latter coefficients to zero the denoised signal is obtained. Two filter lengths: 8 and 20, three levels in the wavelet decomposition and hard thresholding were used. The smoothed data sets are further referred to as W8, W20;

The choice of the filtering methods and their parameters was based on the literature review. The use of approaches earlier applied for eye movement signals, and solutions used in the analysis of other biological signals, was assumed. Hence, the RM choice was driven by the research presented in [22,33], including the selection of window sizes. Similar values for this parameter were applied for the SG filter, for comparison purposes. The polynomial order was grounded in the previously conducted research [34], in which it proved useful for eye movement analysis. In such an application SG was also used in [18,35]. Several works regarding other biosignals such as EEG [36] and ECG [37,38] may be found, too. In the case of the third method, Db wavelets were chosen because of the maximal number of vanishing moments they have. Additionally, their successful usage in the processing of other signals was shown in [39] for electrooculogram (EOG), in [40] for EEG and in [41] for EEG, ECG, and speech. Following the experiments described in the latter research, the medial and maximal filter length were chosen in the presented studies.

Subsequently, each set was transformed to a time series—representing horizontal eye movement velocity obtained by the application of the standard procedure of the two–point signal differentiation. They were subjected to further analysis that consisted of the reconstruction of the system state phase and the LLE estimation as well as the long–term correlation assessment.

All calculations were performed by means of the packages and methods available in the R project environment. For data filtering purposes the following functions were used: (1) the *runmed* method—implementing the RM algorithm—from the *stats* package [42]; (2) the *daubcqf* one was used in order to calculate the wavelet and scaling coefficients for a given filter type; subsequently, they were applied by *densoise.udwt* function in order to denoise signals—both functions are available in *rwt* package [43]; (3) *sgolayfilt* smoothing filter from *signal* package was exploited [44].

Nonlinear dynamics analysis was conducted with the usage of three functions from *tseriesChaos* package [45]: (1) *mutual* in order to calculate the *time delay*; (2) *fnn*—for determining the *embedding dimension*; (3) *lyap*—for system dynamics assessment. Additionally, the long–range correlation was estimated by means of *fractal:DFA* method, which performs a detrended fluctuation analysis and estimates the scaling exponent from the results [46]. The number of intervals for calculating the mutual information was obtained with the usage of *graphics::hist* method, according to Scott’s rule (Equation (7)) [47]:

(7) $$h=\frac{3.5\sigma }{\sqrt[{3}]{n}},$$

where σ is the standard deviation of the distribution and *n* is the number of available samples.

For the statistical analysis of the obtained results the following tests were used: *t*-test [48], Analysis of Variance(ANOVA) [49], Tukey’s Honest Significance Difference(Tukey’s HSD) [50], Kruskal-Wallis rank sum test [51] and the Shapiro-Wilk test of normality [52].

## 3. Results

**Figure 4:**
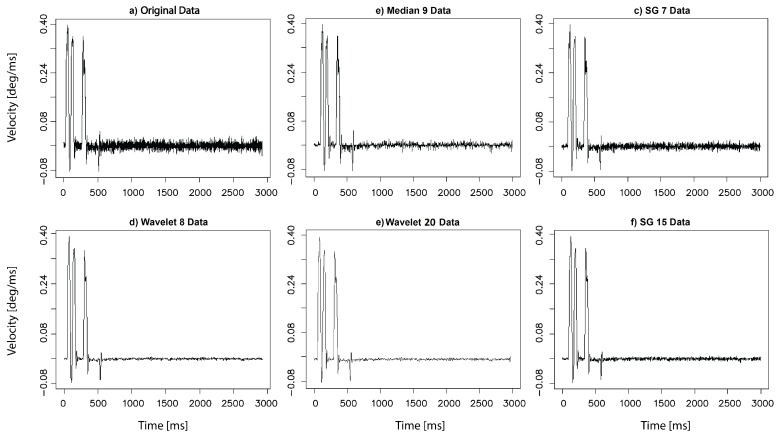
An example time series in the original form (**a**) denoted in text by N and after various filter application: (**b**) M9, (**c**) SG7, (**d**) W8, (**e**) W20 and (**f**) SG15.

The filter application entails, to a certain extent, changes in signal characteristics, which may be easily observed in the example plots presented in Figure 4, prepared for the same time series and for the chosen filtering methods. These changes may have further impact on the parameters used for restoring the system dynamics. This influence was investigated by a comparison of the results evaluated for the original and all filtered time series.

### 3.1. The Phase Space Reconstruction

The first step of the analysis regarded the time delay and embedding dimension—two parameters responsible for the phase space reconstruction. These parameter values averaged over all times series are shown in Table 1. The method for the time delay estimation ensures that its values are not correlated two much. It means that the time delay is: (1) large enough to separate data in the reconstructed phase to provide significantly different information about an underlying system, yet (2) it is not larger than a system memory in order not to lose information about an initial state. Hence, by studying the values in the second column of Table 1, it may be reasoned that the correlation among the recorded samples increases with filter application. It is represented by higher time delay values in contrast to the original time series. This is especially visible when wavelet filters are taken into account. Only SG7–based time series represent similar characteristics to the unfiltered data.

Assessment of the second parameter is shown in the third column of the same table. Similarly, higher values were evaluated for the filtered time series, especially for the RM with larger window sizes and for SG15. This means that for signals smoothed in such a way, higher dimensions are required to unfold orbits from overlapping. It is related to the fact that points taken to define time series, even if separated by higher time delays, are still placed in a close neighborhood for lower dimensions. The data processed by the SG filter with the lower window size, once again obtained similar results to those for the unfiltered data.

Statistical significance of the obtained results was checked and confirmed by means of the Kruskal-Wallis rank sum test at a 0.05 significance level. The choice of the test was driven by the fact that the distribution of both parameter values did not follow normal distribution.

### 3.2. Signal Characteristics Exploration

For further analysis, the time series were divided into several parts—as in the research presented in [14,15]—to make a detailed analysis of eye movement over fixation duration possible. Hence, two main periods were distinguished within a time series: (1) the first 200 ms corresponding to the *saccadic latency*—the time from the moment of the appearance of a stimulus to the commencement of a saccade [53], (2) the second one starting approximately at the time when the fixation point is reached and the eyes remain fixed to obtain scene information. Subsequently, within these two main scopes, smaller sequences were defined:

- three segments for the first period: 0–50 ms, 0–100 ms and 0–200 ms,
- two for the second one: 200–700 ms and 700–1500 ms.

The division introduced in the first period is an extension—in comparison to the earlier conducted studies [14]—and was introduced to learn more about the first milliseconds of eye movement within saccadic latency occurrence.

**Figure 5:**
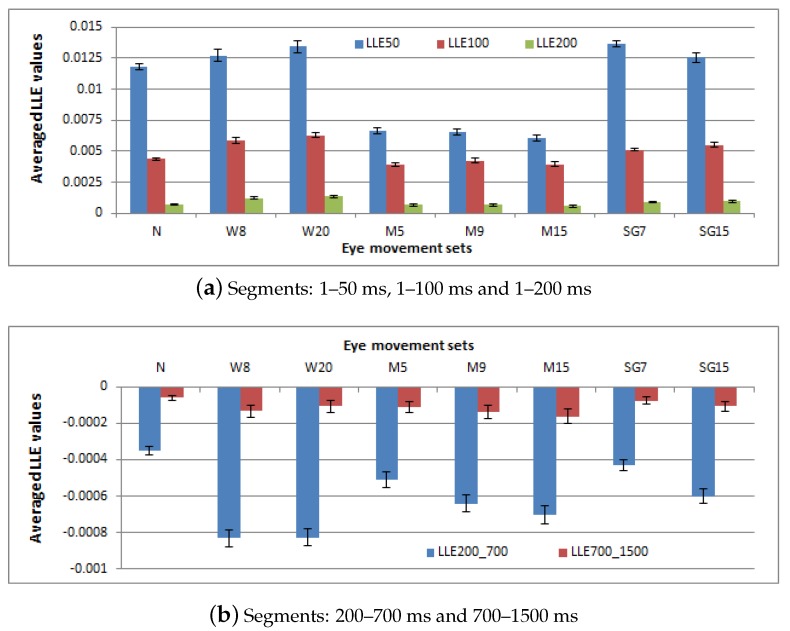
The Largest Lyapunov Exponent (LLE) calculated for different periods of the saccadic latency and for time series processed by different filters.

At first the LLE was evaluated for the sake of examining a chaotic features existence. The calculations were performed for the two main periods independently. The obtained results are shown in two charts in Figure 5—at the top (a) and at the bottom (b)—respectively.

The LLE assumed positive values in all three segments, which is visible in the upper chart: higher at the beginning of the saccadic latency: (0–50 ms) and decreasing with time (0–100 ms and 0–200 ms), but still greater than 0. The influence of the applied filters on the outcomes is visible in the values estimated for particular methods. Similar to the earlier-presented results, the differences related to time series after RM filter application, in which case the lowest strength of chaotic behavior was revealed. Among the other methods, a similar chaotic level was observed; however, the wavelet filter with length equal to 20 and the SG filter with the window length set to 7 revealed the strongest divergence between the time series elements during the system evolution.

As in the case of all time series, eye movement dynamics was represented by very small exponent values, the *t*-test—under the null hypothesis that the LLE value is equal to zero and the alternative hypothesis: *true mean is greater than 0*—was applied. The null hypothesis was rejected with a 95% confidence interval—indicating statistical significance of the results. Corresponding confidence intervals were presented by I-bars on the plot. Additionally, the significance of differences in the results related to different filtering methods was checked. As the Shapiro-Wilk test showed the results were normally distributed, the ANOVA method was used for this purpose—for each period independently. The detailed between-group dependency was explored with Tukey’s HSD test. In the majority of cases, the outcomes turned out to be significantly different; however, in some the null hypothesis was not rejected, as enumerated in Table 2.

A similar analysis was conducted regarding the second main period and its segments: 200–700 ms and 700–1500 ms (Figure 5, at the bottom). This time the LLE values, regardless of the filtering method applied, were negative; indicating convergence towards a stable point or an orbit. A stronger convergence was presented for the first time scope and in the case of wavelet filters. Additionally, greater differences were found among median filters than in the saccadic latency periods. For the second analyzed segment (700–1500 ms), the LLE values were higher, yet still negative, which may stem from two facts:

- the LLE values for the particular time series were indeed higher; the distance between neighbor points was smaller than the initial one, yet did not change with the same rate as in the previous time scope,
- a return to chaotic behavior was observed in the case of a group of time series and, consequently, the occurrence of some positive LLE values influencing the global mean.

To check if the true mean is less than 0, the *t*-test was performed. Its outcomes confirmed statistical significance of these differences and the 95% confidence intervals are presented in the form of I-bars in Figure 5 at the bottom.

When the ANOVA and Tukey’s HSD tests were employed regarding data coming from between 200 and 700 ms—the majority of the compared outcomes turned out to be significantly different. Time series which did not belong to this group are presented in Table 3, in the left column. Once again, the median filters are representatives of these methods, which did not introduce significant differences in the results.

A different situation is observed in the second studied segment—700–1500 ms—where most of the differences in the results turned out to be insignificant, apart from those presented in Table 3 in the right column. They are highlighted in red to enable better differentiation from the opposite cases. Such results may indicate that filters have a greater impact when a system reveals stronger convergence or divergence.

Summarizing all the above-presented results, it must be emphasized that during the system dynamics reconstruction for the number of time series the procedure could not be completed due to the difficulty in the LLE values calculations. This problem was mainly visible in the case of time series which were smoothed with the usage of RM filters. The characteristics of time series after this method application, regardless of the length of the moving window, made the procedure calculating the LLE infeasible, due to the large number of zero values of data series elements. It concerned approximately 35% of M5–sets, 30% of M9–sets and 20% M15–sets. In the case of other filter types, this percentage ranged between 1 and 8, apart from W8, for which it was approximately 12%.

### 3.3. The Phase Space Visualization

**Figure 6:**
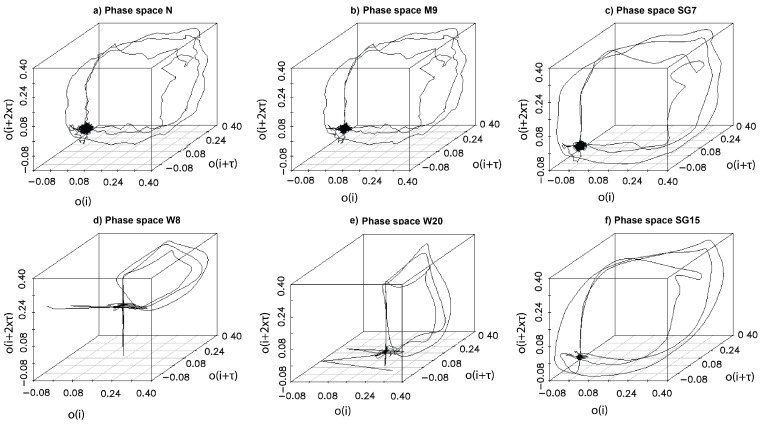
Example phase space plots (orbits) for time series presented in Figure 4. An orbit consists of all points y(i) representing system states. Coordinates of y(i) are calculated from scalar measurements o(i), according to the formula presented in Equation (1). The axes represent coordinates of the first three dimensions of y(i): o(i), o(i+τ), o(i+2τ).

In the previous sections, the nature of eye movement was quantified by various factors such as: time delays, embedding dimensions, and the LLEs. However, the dynamics of a system may also be analyzed in a graphical form, represented by plots of a system phase space. Such a plot is constructed based on Takens vectors [54], a structure which includes coordinates of all points representing system states. Their values are calculated from scalar measurements, according to the formula presented in Equation (1), by means of the previously evaluated parameters: time delay τ and embedding dimension *m*. Analysis of the phase space plot is another tool which may prove useful in ascertaining the influence of the filtering method on system dynamics assessment. When studying examples of the phase space plots drawn for the previously presented time series (Figure 4), shown in Figure 6, it may be perceived that all, bar W8 and W20, follow similar trajectory patterns. Nevertheless, among the remaining group, some differences may also be found. The greatest similarity is visible among the time series created based on the original (N), the median (M9) and SG7-based sets. The time series defined after application of the SG filter (SG7 and SG15) are characterized by smoother lines of the followed path, while the wavelet filters had the greatest impact on the shape of the orbit. All findings are confirmed by the results estimated for these time series (Table 4). For W8 and W20, higher time delay values, stronger chaotic representation (LLE_0_100 values) and stronger convergence (LLE_200_700) were revealed. In the case of the first three earlier-mentioned time series—N, M9, and SG7—similar values for these parameters were estimated. The SG15 set provided the lowest time delay, nevertheless the strength of chaos was exposed at the same level as in the case of W8 and W20. In the last period presented in Table 4, increasing LLE values towards 0 were shown for all six time series.

### 3.4. Detrended Fluctuation Analysis

The second group of tests regarded analysis of the existence of long-term correlation in eye movement signal and for this purpose the previously described DFA method was applied. As stated earlier, according to this method, a time series is divided into intervals and such a division is repeated for various interval lengths. Subsequently, within these intervals the series is detrended by means of a chosen algorithm. Both the algorithm and interval lengths may be set as input parameters of the DFA method. In these studies, two methods: the polynomial of the first degree and bridge detrending—performed by subtracting from the data the line connecting the first and last point of the series—were chosen to be used as the first DFA parameter. For the second one, two groups of different interval scopes, defined by the minimal and maximal length, were used. In both scopes, the minimal value was set to 30, while the maximal was different and equaled either 500 or 1000, which yielded 5 and 6 intervals, respectively. Additionally, prior to the procedure initiation, cumulative summations on each original time series were performed.

The averaged results, calculated for all groups of parameters, together with their standard deviations (placed in brackets) are presented in Table 5. They comprise outcomes for the polynomial and bridge detrending and two different groups of interval lengths and are denoted by: poly:500; poly:1000; bridge:500 and bridge:1000.

When analyzing the results provided in Table 5, minute differences for the same parameter set and for differently filtered time series (rows: 1, 3, 5, 7) may be noticed. Statistical analysis, performed with the usage of the ANOVA test, revealed a lack in their statistical significance. The choice of the ANOVA method was made based on the exploration of the distributions of the results and although the Shapiro test did not confirm their normality, exploration of their density plots justifies such a method selection.

**Figure 7:**
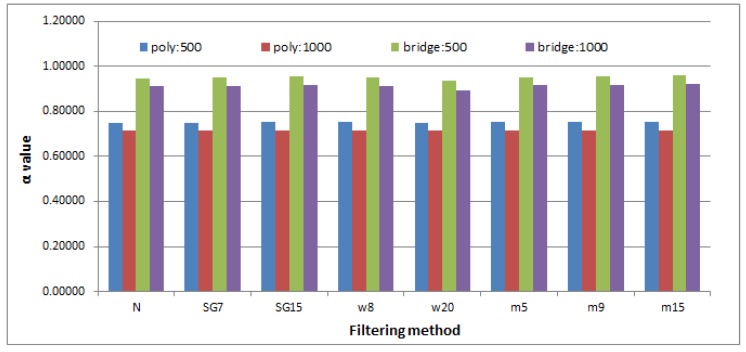
The α values averaged over all time series independently for different smoothing methods and detrended fluctuation analysis (DFA) method parameters.

A comparison of the outcomes, obtained by applying different parameter values (the detrending method or maximal interval length) to time series smoothed with the same method, highlights greater variety (Table 5, columns from N to M15). It is especially visible when the polynomial and bridge detrending are taken into account (Figure 7). Statistical analysis once again was conducted with the ANOVA and Tukey’s HSD tests, yet this time all the differences turned out to be significant.

The above–conducted exploration focused on the DFA results averaged over all time series divided into groups, taking the filtering method as the criterion. Such studies disclose the general characteristic of the whole data set; however, it is also interesting what effect a particular time series had on the final results. It was the motivating factor for inspecting the distribution of α values on the time series basis. For this reason, the results of the DFA for the group of {N, SG7, SG15, W8, W20, M5, M9, M15} data sets were collated separately for each of 1334 time series for comparison purposes. This juxtaposition was aimed at comparing the DFA method results for each original time series (N) and its filtered counterparts to ascertain the influence of a particular filter on long-term correlation assessment. For this reason—within each collated group—the standard deviation was calculated. It was subsequently averaged over all time series for different parameters of the DFA method (poly:500, poly:1000, bridge:500, bridge:1000), independently.

**Figure 8:**
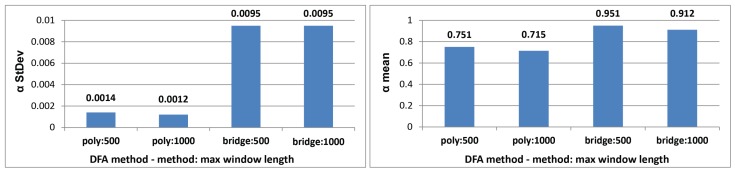
The standard deviation of the α parameter (on the **left**) and its mean values (on the **right**), calculated separately for each time series based on its corresponding sets: N, SG7, SG15, W8, W20, M5, M9, M15 and averaged over all 1334 time series, for different parameters of the DFA method, independently.

These values, shown in Figure 8 on the left-hand side, revealed a small dispersion of α parameter, which is especially visible in the case of the polynomial detrending method. On the right-hand side in Figure 8, the α mean values are presented to facilitate the analysis of the standard deviation.

**Figure 9:**
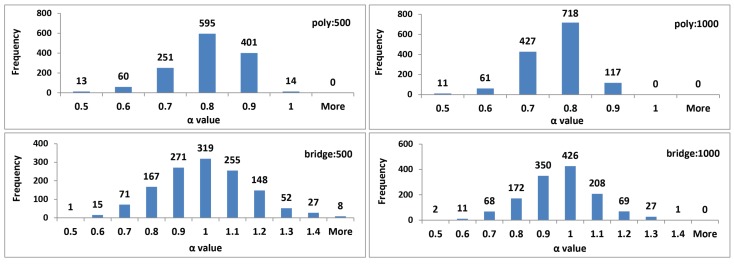
The histogram of α values prepared for the chosen filtering method—SG7—and different parameters of the DFA method.

Additionally, the distribution of the α parameter values obtained for all 1334 analyzed time series, for one chosen smoothing method—the SG filter with window length equal to 7—and for different parameters of the DFA method were prepared (Figure 9). This data set may be treated as the representative one, based on analysis of information disclosed in Figure 8. It points out a similar density distribution of α values independently on the filter applied when the same set of the DFA method parameters is used.

**Figure 10:**
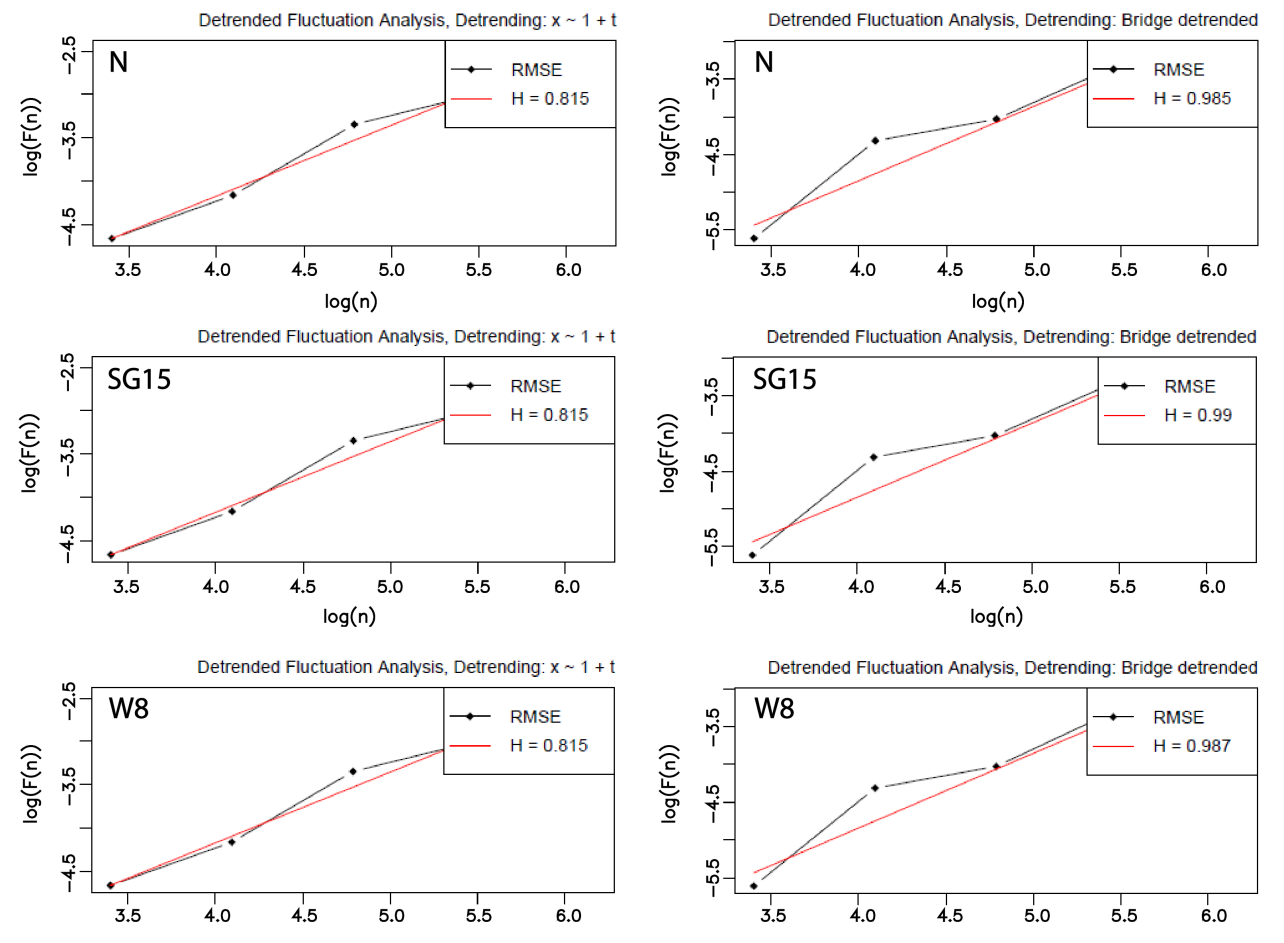
Example double logarithmic plots representing the fluctuation F(n) in the function of *n* (denoting the interval length) for the chosen time series. The left column shows polynomial detrending with the maximal *n* set to 500 and three different types of smoothing—without any (N), SG15, and W8. The right one represents the same data sets, with the same n scope and the bridge detrending method. RMSE: root mean square error.

Furthermore, the example double logarithmic plots for one chosen time series and selected, different parameters of the DFA method are shown in Figure 10. The left column presents charts for the polynomial detrending with the maximal length interval set to 500, while on the right, the same interval size was applied with the usage of the bridge detrending. The first row of the charts relates to the original time series, the second one to the time series pre-processed by the SG filter with the window size set to 15 and finally, in the third row, plots for W8–based sets are included.

## 4. Discussion

The presented studies were aimed at both the assessment of several eye movement features and ascertaining how a preprocessing method may influence the unveiling of these features.

The dynamics of eye movement signal has been earlier explored, based on the eye positions in [12] and its velocity in [14,15], and in all these cases chaotic behavior was revealed. Thus, this research concentrated on the impact the applied filter may have on the classification of a signal dynamics. Studying the results presented in Figure 4, Figure 5 and Figure 6, as well as in Table 1, it may be noticed that they provide evidence of the existence of such an impact, although the representation of the overall eye movement behavior turned out to be similar regardless of the filtering method used. This influence was especially visible in the estimated phase space parameters. The time delay—used for separating correlated measurements, and embedding dimension—ensuring unfolding orbits from overlapping, revealed different values for the different methods applied. Removal of some frequency bands, as well as smoothing, also introduced changes to the chaos strength; however, this did not influence its nature in the analyzed time periods.

For all filtering methods, the periods between 0 and 200 ms were characterized by the chaotic behavior of corresponding strength. In the cases of the segments: 200–700 ms and 700–1500 ms, although the opposite (to the first 200 ms) LLE characteristic was revealed, this was however similar between the filtered groups as well as within particular time sections. Thus, taking all these results into account, it may be reasoned that the application of filters for improving data quality does not affect the data in such a way that changes the underlying system dynamics. An additional argument for such a conclusion may be found in [14], where, for eye movement dynamics reconstruction, a low-pass filter with a 50 Hz cut-off frequency was used. There was the similar signal nature exposed. However, it must also be emphasized that the used filter method may influence the process of ascertaining system behavior, which was encountered in the case of median filters and the LLE value estimations.

The second part of this research had two purposes—(1) to check the existence of long–range correlation in eye movement signals and (2) to verify how its strength depends on the smoothing method used. The outcomes obtained for the analyzed time series in the first step revealed the presence of a long-range correlation that represents fractal characteristics expressed by self-similarity. Furthermore, they showed, as expected, the impact of the various range of parameters used on the DFA method results. It is exposed by the different values of the parameter α obtained for different detrending algorithms and interval scopes. When studying the contents of Table 5 and Figure 7, it may be noted that the highest values were provided by the *bridge detrending* method and interval lengths belonging to the scope ranging from 30 up to 500 ms. Slightly lower values were achieved for the same method, when the scope of intervals was lengthened to 1000 ms, meaning that the correlation measured over longer times series segments weakens to some extent. A similar dependency may be observed in the case of the polynomial method; however, when comparing its results to the first method, generally lower values were received.

The interesting finding noticeable in both Table 5 and Figure 7, is the almost invisible influence of the applied filters on the α parameter estimates. Additionally, low standard deviations in the results indicates small differences between values calculated for time series belonging to the particular groups of filtered data: N, SG7, SG15, W8, W20, M5, M9, and M15 sets. This finding confirms the statement that DFA is a well-established method for determining the scaling behavior of noisy data in the presence of trends without knowing their origin and shape [29].

A comparison of the outcomes evaluated for each time series independently, when taking its *smoothed-by-various-filters* counterparts into account, leads to the same conclusion (Figure 8). Low values of the standard deviation obtained for each time series and then averaged for over all time series, highlight the tendency of the results to be close to the mean, wherein greater dispersion is visible for bridge detrending (Figure 9, charts at the bottom). The results provided for this method also revealed some percentage of time series for which α values are greater than 1. It indicates the existence of long-range correlation, but such that ceases to follow the power-law rules. The value reaching 1.5 indicates Brownian motion [55] and the Hurst exponent should be calculated as H=α−1. However, such a situation is not present when the upper charts in Figure 9—corresponding to polynomial detrending—are analyzed. For all time series α values lower than 1 were gathered. These values are comparable for those presented in [12], although they were evaluated only for one person.

Discrepancies in the results obtained for different parameters of the DFA method show that usage of several different approaches is required to describe characteristics of the eye movement signal as closely as possible. Such studies are planned as future research directions, among which usage of the Hilbert-Huang Transform [56] and analysis of Higuchi’s fractal dimension [57] are considered. Additionally, some limitation of these studies may be pointed out. The first one is related to the usage of filtering methods and the choice of their parameters. Both were inspired by the literature review and similar set-ups were used for all participants. If examinations were focused on a particular eye movement system, an adjustment of the parameters would be required. Moreover, for the purpose of this experiment, one stimulus type was applied. It would also be valuable to repeat the presented examination using other stimuli kinds, which is planned as a further investigation, too.

## 5. Conclusions

One of the purposes of the presented studies was to uncover dynamical and fractal features of eye movements to understand oculomotor system behavior based on the signals registered for young people with normal vision. By revealing chaotic and self-similarity characteristics, the results confirmed preliminary expectations regarding the explored system’s nature. Based on this paper’s findings it has become possible to commence comparative studies aimed at providing such a description for people affected by various diseases.

The second goal of these studies—analysis concerning the effect the applied filter has on the representation of eye movement characteristic—showed that although such preprocessing influences the final results to a certain degree, it does not change the system’s general behavior assessments. The selection of the input parameters turned out to be more significant for the methods used. The differences in the outcomes obtained for this part of the research provide arguments for further studies in this field.

## Figures and Tables

**Table 1:** Estimations of the time delay and embedding dimension, averaged over all time series. Standard deviations are placed in brackets.

Filters	Time Delay	EmDim
N	9.483 (10.988)	4.143 (2.638)
W8	21.149 (12.693)	6.388 (6.222)
W20	19.333 (10.922)	6.247 (6.156)
M5	13.164 (7.940)	6.698 (4.544)
M9	14.560 (6.800)	7.591 (5.185)
M15	15.765 (6.529)	8.324 (5.727)
SG7	9.500 (9.952)	3.994 (3.118)
SG15	15.945 (10.817)	7.143 (7.884)

**Table 2:** Time series, between which significant differences in the LLE values were not confirmed—the first main period (0–200 ms).

Segment		
0_50	0_100	0_200
M5:M9	M5:M15	M5:M15
W8:SG15	N:M9	M9:M15
W20:SG7		N:M15
		SG7:SG15

**Table 3:** Time series, between which significant differences in the LLE values were not exposed (200–700 ms)—on the left and those for which significant differences were confirmed (700–1500 ms)—on the right.

Segment	
200_700	700_1500
N:SG7	N:W8
M9:M15	N:M9
SG7:M15	N:M15
SG15:M9	SG7:M15

**Table 4:** Results obtained for the time series presented in Figure 6 during the underlying dynamics reconstruction.

Filter	Time Delay	emDim	LLE_0_100	LLE_200_700	LLE_700_1500
N	10	3	0.00830	−0.00082	−0.00002
M9	10	5	0.00960	−0.00151	−0.00009
SG7	10	3	0.00964	−0.00096	−0.00002
SG15	8	3	0.01189	−0.00138	0.00000
W8	17	3	0.01349	−0.00178	−0.00004
W20	19	4	0.01031	−0.00187	−0.00001

**Table 5:** The α values averaged over all time series for all parameter sets. Standard deviations are placed in brackets.

Param Set	N	SG7	SG15	W8	W20	M5	M9	M15
poly:500	0.74962	0.75063	0.75201	0.75102	0.75022	0.75127	0.75194	0.75299
	(0.0853)	(0.0841)	(0.0831)	(0.0841)	(0.0839)	(0.0837)	(0.0833)	(0.0829)
poly:1000	0.71396	0.71476	0.71589	0.71510	0.71444	0.71528	0.71528	0.71675
	(0.0692)	(0.0682)	(0.0672)	(0.0681)	(0.0679)	(0.0678)	(0.0678)	0.0670)
bridge:500	0.94499	0.94812	0.95531	0.95194	0.93809	0.95116	0.95428	0.95859
	(0.1690)	(0.1652)	(0.1628)	(0.1628)	(0.1533)	(0.1643)	(0.1640)	(0.1636)
bridge:1000	0.90989	0.91238	0.91814	0.91077	0.89468	0.91479	0.91734	0.92089
	(0.1362)	(0.1331)	(0.1309)	(0.1312)	(0.1236)	(0.1323)	(0.1320)	(0.1315)

## Supplementary Materials

Data set used in the research is available online at http://kharezlak.pl/eyetracking.html.
